# Impact of SARS-CoV-2 Infection in Children with Asthma and Impact of COVID-19 Vaccination: Current Evidence and Review of the Literature

**DOI:** 10.3390/microorganisms11071745

**Published:** 2023-07-04

**Authors:** Roberto Grandinetti, Elisabetta Palazzolo, Luisa Rizzo, Roberta Carbone, Giovanna Pisi, Valentina Fainardi, Susanna Esposito

**Affiliations:** Pediatric Clinic, Department of Medicine and Surgery, University of Parma, 43126 Parma, Italyelisabettapalazzolo24@gmail.com (E.P.); gpisi@ao.pr.it (G.P.); valentina.fainardi@unipr.it (V.F.)

**Keywords:** asthma, COVID-19, COVID-19 vaccines, fragile children, SARS-CoV-2

## Abstract

The clinical aspects of SARS-CoV-2 infection, as well as the COVID-19 vaccines’ safety, efficacy and effectiveness in pediatric patients with asthma, are crucial to adapting clinical management in this fragile population and for prevention strategies. The aim of this narrative review was to evaluate the impact of SARS-CoV-2 infection in children with asthma and the impact of COVID-19 vaccination. Systematic research using the principal medical databases was conducted using specific search query strings from the early spreading of COVID-19 globally until March 2023; further relevant data were drawn from the main national and supranational institutions. No significant differences in SARS-CoV-2 incidence and morbidity were found in asthmatic pediatric patients compared to non-asthmatic ones; however, subjects with uncontrolled asthma were found to be at increased risk of developing a serious disease during SARS-CoV-2 infection. Regarding COVID-19 vaccines, accumulating data support their safety, efficacy and effectiveness on asthmatic children regardless of asthma severity. Further cohort-based studies are needed as the evidence of new epidemic waves caused by new viral variants makes the current knowledge outdated.

## 1. Introduction

Since its initial spreading from the first months of 2020 to the present day, more than 740 million cases of COVID-19 have been recorded all over the world, with over 7.5 million deaths among the general population, with nearly half of these latter in Europe and North America [1]. The pediatric population seems to be less affected by COVID-19 than other age groups, both in terms of rates of infection by SARS-CoV-2 [2] and also in terms of clinical courses with less severe cases and lower rates of hospitalization and death [3]. Several reasons have been given to explain these differences, including a generally lower susceptibility to the infection, lower case detection due to the higher prevalence of pauci- or asymptomatic cases and also the presence of more immature respiratory and immune systems in the presence of minor or no comorbidities [4,5].

The rapid development of COVID-19 vaccines made by the extraordinary efforts of the pharmaceutical industry and national and supranational regulators agencies and their subsequent extension to the pediatric population was a crucial element against the pandemic, especially for the category of frail subjects. Among these, it was initially debated whether the asthmatic pediatric population could be considered or not as a fragile category, because only a few studies have reported asthma as a risk factor for severe forms of COVID-19 [6,7,8,9,10]. Actually, both the clinical aspects and those related to the vaccines’ immunogenicity in specific cohorts, including the asthmatic population (since SARS-CoV-2 infections involve mainly, but not exclusively, the respiratory tract), are crucial aspects in better understanding how COVID-19 can be more effectively fought. The aim of this narrative review is to describe these aspects in the pediatric population affected by asthma by a critical review of the current literature.

## 2. Materials and Methodology

Systematic research on the principal databases (PubMed, Scopus and the Cochrane Library) was conducted using specific search query strings (see the Supplementary Materials), with a time interval from the early spreading of COVID-19 globally until 31 March 2023. More than 1700 articles were found and screened and subsequently included in this review. In addition to the above-mentioned workflow, further relevant data were drawn from the main national and supranational institutions (i.e., WHO, FDA, EMA and CDC) concerning the COVID-19 vaccination issue and also extracted from the guidelines and position papers of specialized scientific societies.

## 3. Incidence of SARS-CoV-2 Infection and Severity of Disease in Pediatric Population with Asthma

### 3.1. Epidemiology of COVID-19 in Patients with Asthma

Comorbidities are known to be a risk factor for severe COVID-19. In a retrospective study including 177 children, 63% of those hospitalized with COVID-19 had comorbidity conditions compared to 32% of non-hospitalized ones with COVID-19, and 78% of critically ill children with COVID-19 had underlying conditions compared to 57% of hospitalized, non–critically ill patients with COVID-19 [7]. In another research, it was observed that, out of 48 children admitted to the pediatric intensive care unit (PICU) for COVID-19, 83% had an underlying disease [8]. In a cross-sectional study, Kara et al. demonstrated that, among 292 pediatric patients hospitalized, 21.2% had a preexistent comorbidity, with obesity (5.1%) and bronchial asthma (4.1%) being the most common underlying diseases [9]. In a cross-sectional study of more than 43,000 children with a COVID-19 diagnosis, it was shown that asthma was the most common diagnosed comorbidity (10.2%), followed by neurodevelopmental disorders (3.9%), anxiety and fear-related disorders (3.2%), depressive disorders (2.8%) and obesity (2.5%) [10].

Asthma is the most common chronic disease of childhood in the world, and asthma exacerbation is frequently caused by viral infections [11,12]. Initially, asthma patients were classified as an at-risk group for COVID-19 severe forms, but whether asthma has worse COVID-19 consequences is not yet clear [13]. However, some research has suggested that there is no apparent heightening of asthma-related morbidity in children with well-controlled asthma [14]. It has been hypothesized that this phenomenon could be associated with lockdown restrictions, school cancellations and social distancing, limited children’s physical activity and reduced exposure to environmental triggers [15]. In a recent meta-analysis, there was no data about a higher risk for severe COVID-19 in asthmatic children, but evidence was lacking [16].

Further studies have reported that young patients with well-controlled asthma did not develop severe COVID-19, probably due to low angiotensin-converting enzyme 2 (ACE2) expression in bronchial epithelial cells [17,18]. ACE2 is the receptor that permits SARS-CoV-2 and spike protein cell entry [18]. There are several data concerning that the type of asthma inflammation may be an important factor in the risk of SARS-CoV-2 infection. [19]. In the pediatric population, asthma is mainly characterized by type 2 inflammation, which is determined by the dominance of T helper 2 (Th2) cells, their hallmark cytokines (i.e., IL-5 and IL-13) and eosinophilia [18]. IL-13 inhibits the expression of the SARS-CoV-2 host cell entry ACE2 receptor on airway epithelial cells; diminished levels of ACE2 transcripts have been associated with high IgE levels and allergen exposure [20]. Thus, asthma may also be protective as the ACE2 receptor, important for SARS-CoV-2 infection and might be underexpressed in the lungs of atopic children [21]. Indeed, ACE-2 was found to be decreased in patients with allergic asthma or in those receiving inhaled corticosteroids [22]. Kimura et al. demonstrated that, in asthmatic and atopic patients, Th2 inflammation decreases ACE2 and increases TMPRSS2 gene expression in nasal and bronchial epithelial cells [23]. TMPRSS2 is a crucial gene in the pathogenesis of SARS-CoV-2 infection, since it produces the protein that cleaves the spike protein into two subunits, allowing viral fusion with the cell membrane [24]. Moreover, there is some evidence demonstrating that inhaled corticosteroid therapy is also associated with a reduction in ACE2 and TMPRSS2 gene expression from sputum [25]. These data show the importance of maintaining asthma control using treatment according to the best practices, including inhaled corticosteroids.

### 3.2. Clinical Course and Severity of COVID-19 in Patients with Asthma

Although, in adults, the current evidence shows that asthma is associated with a higher risk of COVID-19-related hospitalization and mechanical ventilation [25], few studies have analyzed this relationship in children, and the results in the literature are still ambiguous [26,27]. In a large multicenter study evaluating COVID-19 disease severity in more than 19,000 patients presenting for emergency care at US pediatric hospitals, it was found that asthma increased the risk for hospitalization (adjusted odds ratio (aOR), 1.4; 95% confidence interval (CI), 1.3–1.6) but not the severity of the disease among those hospitalized [28]. The same results were found in other studies. A case–control study was conducted comparing three groups: 142 children with COVID-19 and asthma, 1110 children with COVID-19 without comorbidities and 140 children with asthma without COVID-19 [29]. In the last group, asthma was identified as a risk factor for hospitalization in children with COVID-19 but not for worse clinical outcomes; asthma exacerbation was not plausibly triggered by SARS-CoV-2 infection, and the degree of asthma severity did not correlate to a higher risk of COVID-19 [29]. Beken et al. studied the factors that impact hospitalization in patients with COVID-19, and none of the allergic conditions, including asthma, were identified to be a risk factor for hospitalization [26].

Regarding clinical presentation, no significant differences were found between asthmatic and non-asthmatic children with COVID-19. The Global Asthma Network (GAN) conducted a global survey at 14 GAN centers from 10 different countries, collecting data between November 2020 and April 2021 [30]. Overall, 169 children with asthma infected by SARS-CoV-2 were included. They found that about 90% of asthmatic children were asymptomatic or had mild COVID-19, the proportion of symptomatic cases increased with age and adolescents were less likely to be asymptomatic than younger children. Compared with previous reports on COVID-19 in children, this study revealed that asthmatic children did not appear to have a higher frequency of severe COVID-19 [30]. Nursoy et al. compared the COVID-19 disease course among 89 children with asthma and 84 healthy peers [13]. The two groups had similar clinical features: degree of hospitalization, percentages of symptomatic disease and fever duration. For example, 96.6% of the asthmatic COVID-19 patients, as well as 92.9% of the healthy children, were symptomatic, with no significant difference between them. Dyspnea (from mild to moderate forms) was reported more significantly in asthmatic COVID-19 cases in comparison to the control group (10% and 1.2%, respectively; *p* = 0.012), but no significant difference was found concerning the other symptoms. However, there was no severe asthma case in this study group, and the authors concluded that asthmatic children with mild to moderate forms experienced COVID-19 with no great differences compared to their peers [13].

Asthma control plays a central role in this category of children. A Scottish cohort study was done on all children from 5 to 17 years of age who were included in a specific dataset (EAVE II, Early Pandemic Evaluation and Enhanced Surveillance of COVID-19) [31]. The aim of the study was to assess the risk of hospitalizations related to COVID-19 in a cohort with poor-controlled asthma: asthmatic children without previous hospital admission for asthma were at an increased risk of COVID-19 hospital admission compared to non-asthmatic children; children with previous hospital admission for asthma were at an increased risk of COVID-19 hospital admission compared to the other groups analyzed. Notably, the use of two different oral corticosteroids as a marker of poor asthma control was linked to an increased risk. This study found that children with poorly controlled asthma had higher rates of COVID-19 hospital admission compared to those with well-controlled asthma or without asthma [31]. This analysis stressed the importance of maintaining good asthma control and the careful monitoring of children with poorly controlled asthma if they develop SARS-CoV-2 infection. Good asthma control can help to protect children from developing more severe manifestations of COVID-19.

The hyperinflammation status is strictly linked with the severity and complications of SARS-CoV-2 infection [19]. In asthmatics, such hyperinflammation can be downregulated by several mechanisms: the eosinophils protective role on the airways, delayed and ineffective antiviral responses due to lower interferon (IFN)-α production by dendritic and epithelial cells and the use of inhaled steroids and their immunomodulatory and antiviral features [28]. Interestingly, according to this argumentation, such mechanisms are responsible for a protective effect during SARS-CoV-2 infection while contributing to a higher morbidity and worse lung involvement in most of the other respiratory viral infections in asthmatics [24].

Summing up, asthma represents a frequent comorbidity in hospitalized children with COVID-19, but the infection has a similar clinical course in patients with mild and moderate asthma compared to non-asthmatic ones. On the other hand, patients with severe and uncontrolled asthma are at a higher risk of developing a serious disease. Maintaining good asthma control is fundamental in the prevention of this risk. Further investigations are required to establish the real impact of asthma on the morbidity and mortality of COVID-19 in the pediatric population.

### 3.3. Management of Children with Asthma and SARS-CoV-2 Infection

Multiple guidelines on allergy care during the COVID-19 outbreak support that asthmatic children should continue with their current asthma medications while infected with COVID-19 (inhaled corticosteroids, long-acting bronchodilators, antileukotrienes and, if necessary, oral corticosteroids) if a good control for symptomatology has been reached [32]. Reducing or suspending the use of controller asthma medications could worsen asthma control and increase the risk of severe exacerbations. Concerning biologic agents (e.g., Omalizumab or Mepolizumab) that are approved for moderate-to-severe asthmatic forms in the pediatric population, the current recommendation is to continue with their administration, since there is no proof that they augment the risk of COVID-19 infection or morbidity. A unique exception may be the interruption of their administration during the acute phase of SARS-CoV-2 symptomatic infection [32]. Aeroallergen immunotherapy (AIT) can be effective as an add-on therapy in the maintenance of asthma control [33]. The administration of AIT has decreased throughout the pandemic (for local or generalized lockdown and also because a more pronounced transition to telemedicine has begun), so home immunotherapy administration has become a valid option, both safe and cost-effective; these implications will endure throughout the pandemic and in the future [34].

Regarding asthma exacerbation, prompt treatment with oral corticosteroids in moderate-to-severe asthma exacerbation is recommended, especially if patients are poorly responsive to bronchodilators, as indicated by the current guidelines [6,21]. In the first phase of the COVID-19 outbreak, oral corticosteroids were not recommended due to their supposed role in immune depression against viral infections. Afterwards, many studies showed the beneficial effects of these drugs in reducing acute respiratory distress syndrome and systemic inflammation [35]. For asthma exacerbation, oral corticosteroids given in a short course are recommended to prevent severe outcomes according to clinical judgment [24]. In a very few and rare cases, patients with severe forms of asthma requiring long-term treatment with oral corticosteroids should be continued with the lowest possible dose to reduce the risk of severe exacerbation. In this cohort, an add-on biologic therapy should also be considered (due to their proven steroid-sparing effect) [24].

If a child is using a nebulized asthma relief medication, this should be switched to a metered dose inhaler (MDI) or dry powder inhaler under most circumstances [36]. Nebulization increases the risk of infection transmission (caused by a higher stimulation of coughs but also by the generation of a higher volume of respiratory aerosols); a lower lung deposition of viral particles has also been proven. Nebulization should only be considered for asthmatic patients unable to use MDI correctly or in cases of medication shortages. Avoiding the respiratory function test (spirometry above all) in patients with confirmed/suspected infection by COVID-19 is also recommended, due to the viral shedding that may expose either the medical staff or other patients to the infection (these tests should be postponed or performed only in cases of necessity) [24]. An increase in telemedicine has been sponsored during COVID-19, especially for children with mild-to-moderate asthma (i.e., well controlled over the past 6 months, with no record of emergency room visits and ≤1 oral steroid bursts) [37]. Optimizing asthma control is effective when the correct management of comorbidities is implemented; above all, rhinitis has been relevant during the COVID-19 pandemic, since its poor control may mimic viral infection with a possibly augmented risk of viral shedding [24]. Other recommendations in obtaining good control over asthmatic symptoms include avoiding well-known asthma triggers (aeroallergens), physical distancing, frequent handwashing and a routine examination of inhaler technique [21,32].

Table 1 summarizes the main recommendations for the management of asthmatic pediatric patients with COVID-19.

## 4. Efficacy and Effectiveness of COVID-19 Vaccines in Pediatric Patients with Asthma

### 4.1. COVID-19 Vaccines in Pediatric Population

In January 2020, the first genetic sequence of the SARS-CoV-2 virus was published [38]. Since then, scientists around the world have collaborated to develop effective vaccines against COVID-19. The first vaccines that were approved globally at the end of 2020 for the adult population were the mRNA-based COVID-19 vaccines Comirnaty (BNT162b2) and Spikevax (mRNA-1273), produced, respectively, by the pharmaceutical companies BioNTech/Pfizer and Moderna [39]. After approximately 12 months, the indication of these vaccines has been extended to the pediatric population [40,41,42].

COVID-19 vaccines are safe, as seen in the registration trials and as also witnessed by the supranational pharmacovigilance systems with their regular reports [43]. The two mRNA-based vaccine preparations differ in terms of storage method in relation to the lipid nanoparticles of which the vaccines are composed that allow drug delivery to human cells [44]. Substantially, Spikevax can be stored at temperatures of +2/+8 °C for 30 days, while Comirnaty requires a storage temperature of −70°/+10 °C; in the first case, in fact, some of the lipids that make up the lipid nanoparticles (in particular, SM-102 lipid) make the vaccine more “stable” and therefore capable of being stored in refrigerators for 30 days, because it is less sensitive to temperatures.

The registration trials for pediatric populations have shown excellent safety, immunogenicity and efficacy profiles [45,46,47,48,49]. However, the vaccine efficacy and effectiveness in real life have changed as a result of the circulation of new viral variants [50,51]. This explains the slight decrease in efficacy compared to the registration trials, although the data even in real life must absolutely encourage the implementation of vaccination strategies for those of pediatric ages [52,53]. It is therefore imperative to design new studies that take into account the epidemiological situation concerning viral variants and specific groups of individuals, including fragile categories. The pediatric asthmatic population could be an excellent study group in terms of numerosity; biological characteristics and evaluation of measurable clinical outcomes (i.e., frequency of exacerbations, therapeutic changes and impact on the emotional–behavioral sphere).

### 4.2. COVID-19 Vaccines in the Pediatric Asthmatic Population

Available data regarding COVID-19 vaccines in asthmatic pediatric patients are few. Recommendations on times and doses of administration do not vary compared to other subjects of the same age; in addition, there is no evidence to support the hypothesis that patients with asthma, regardless of their age, are at increased risk of exacerbation when receiving the COVID-19 vaccination [54]. In addition, therapy routinely used in bronchial asthma (i.e., inhaled corticosteroids and leukotriene receptor blockers) does not have an impact on the immunogenicity of COVID-19 vaccines, so there is no need to interrupt these medications on the day of vaccination or the surrounding days [55], unlike for biological therapies, for which an interval of 2–7 days between the administration of the COVID-19 vaccine and their administration is necessary (this theme will be addressed in a separate section). Concerning the risk of possible allergic reaction, unless the patient has a known previous reaction to some specific component of the vaccine, there are no contraindications to administering COVID-19 vaccines, since the registration trials and post-market surveillance have widely proven their safety [56].

Greater attention must be paid to subjects with uncontrolled asthma, since these patients are considered at moderate risk of adverse reactions [57]. In accordance with scientific societies guidelines, before carrying out vaccinations on these subjects, a stabilization of the clinical conditions must be done [40,58]. For those suffering from severe persistent bronchial asthma well controlled by therapy, the vaccination is recommended alongside a prolonged observation period of 60 min [40,58]. In cases of uncontrolled asthma, the administration of the vaccine should be postponed until the clinical situation is under control again. Since the main cause of uncontrolled asthma is nonadherence to proper therapy, it is important to direct the patient to a qualified center. If asthma control is not achieved despite optimal therapy, vaccination in a protected environment (i.e., hospital) with observation for 60 min should be recommended [40,59].

One further aspect on which it is important to focus attention toward is vaccine hesitancy, since many parents have been hesitant to vaccinate their children with asthma [60]. Studies have been carried out in order to understand the perplexities and the sources of this indecision, mainly by a comparison with studies on vaccination sentiment against influenza [61,62]. The results of these studies claimed that advice from their pediatrician was an important factor in overcoming vaccine hesitancy, whereas, surprisingly, the level of asthma control did not appear to be a significant factor in the parental decision process [62,63]. A higher level of parental education represents a factor favoring the decision to vaccinate children for both influenza and COVID-19; studies have also confirmed that subjects who had previously received an influenza vaccination had a greater intention to receive a COVID-19 vaccination [52,53,64]. This information highlights that, to reach high vaccination coverage against COVID-19 among pediatric asthmatic patients, optimal communication should involve the whole family and the pediatricians who are routinely in charge of the patients.

### 4.3. COVID-19 Vaccines in Pediatric Patients with Severe Asthma Treated with Biological Therapies

Biological drugs can block specific immune pathways that limit the cascade determining inflammation, and for this reason, they are used in the treatment of severe forms of asthma [65]. Many monoclonal antibodies are currently approved for severe type 2 asthma in adults: omalizumab, mepolizumab, benralizumab, reslizumab and dupilumab [66]. In pediatric ages, the first two have been approved from 6 years of age up, but the last three can only be administered off-label [66]. Since asthma (along with other atopic diseases) does not increase the risk of an allergic reaction to COVID-19 vaccines, treatments with biological drugs should not be stopped [9,30], but it is recommended not to receive a COVID-19 vaccine on the same day as an injection of biologics [40,55,59]. One study showed no differences in immunologic responses against COVID-19 infection in asthmatic patients treated with biologics compared to patients who did not take such therapies, encouraging COVID-19 vaccination in this fragile category [63]. A precaution to follow is to distance the COVID-19 vaccination from the administration of biological therapy about 2–7 days [40].

In conclusion, in severe asthmatic pediatric patients, COVID-19 vaccines are strongly recommended alongside basic hygienic measures and the use of personal protective equipment [67].

Table 2 summarizes the recommendations for COVID-19 vaccinations in asthmatic pediatric populations.

## 5. Conclusions

SARS-CoV-2 infection has a similar clinical course in patients with mild and moderate asthma compared to non-asthmatic ones. However, patients with severe and uncontrolled asthma are at a higher risk of developing a serious disease, so maintaining good asthma control is fundamental in the prevention of this possibility. These strategies could be implemented with close cooperation between pediatricians, patients and their families, explaining the importance of accurate treatment compliance, the necessity of periodic medical evaluations and providing them a written action plan with instructions on how to manage the reappearance or worsening of symptoms. COVID-19 vaccines are recommended in pediatric patients with asthma, who should continue to receive their prescribed asthma medications on the day of their COVID-19 vaccination or on the surrounding days. For subjects with severe persistent bronchial asthma, an observation of about 60 min after administration of the COVID-19 vaccine is required, whereas an interval of 2–7 days between the administration of the COVID-19 vaccine and biological therapy is necessary. However, further specific population studies that take into account the epidemiological peculiarities of this category of patients are needed to better understand the underlying causes of differences with other fragile population segments and to better act against the pandemic. In addition, further cohort-based studies are needed, as evidence of new epidemic waves caused by new viral variants makes the current knowledge outdated.

## Figures and Tables

**Table 1:** Management of the asthmatic pediatric population with COVID-19: tips and recommendations.

SARS-CoV-2 infection has a similar clinical course in patients with mild and moderate asthma compared to non-asthmatic ones.
Patients with severe and uncontrolled asthma are at a higher risk of developing a serious disease, so maintaining good asthma control is fundamental in the prevention of this possibility.
The controller medications should not be stopped or reduced, since these therapeutic changes can worsen asthma control and increase the risk of a severe exacerbation.
Biologic agents should be continued; there is no current evidence about their implications in a higher risk of COVID-19 infection or a higher mortality or morbidity.
A suspension of biologic agents during the acute phase of COVID-19 infection can be allowed, in case of other infectious events.
Considering asthma exacerbations, a short course of oral corticosteroids can be administered to prevent severe outcomes according to clinical judgment.

**Table 2:** COVID-19 vaccination in asthmatic pediatric population: tips and recommendations.

There are no contraindications to the administration of COVID-19 vaccines to asthmatic patients.
Advise patients to continue taking their prescribed asthma medications (i.e., inhaled corticosteroids and leukotriene receptor blockers) on the day of the COVID-19 vaccination or on the surrounding days.
Subjects with uncontrolled asthma are considered at moderate risk of an adverse reaction; it is necessary to stabilize the clinical conditions.
For subjects with severe persistent bronchial asthma, an observation of about 60 min after administration of the COVID-19 vaccine is required.
An interval of 2–7 days between the administration of the COVID-19 vaccine and biological therapy is necessary.

## Data Availability

Not applicable.

## Supplementary Materials

The following supporting information can be downloaded at: https://www.mdpi.com/article/10.3390/microorganisms11071745/s1, Supplementary material S1: Clinical questions and PICO items.
